# *Stephania japonica* Ameliorates Scopolamine-Induced Memory Impairment in Mice through Inhibition of Acetylcholinesterase and Oxidative Stress

**DOI:** 10.1155/2022/8305271

**Published:** 2022-02-21

**Authors:** Md. Yusuf Al-Amin, Amitav Lahiry, Rafia Ferdous, Md. Kamrul Hasan, Md. Abdul Kader, AHM Khurshid Alam, Zahangir Alam Saud, Md. Golam Sadik

**Affiliations:** ^1^Department of Pharmacy, University of Rajshahi, Rajshahi 6205, Bangladesh; ^2^Department of Biochemistry and Molecular Biology, University of Rajshahi, Rajshahi 6205, Bangladesh

## Abstract

Alzheimer's disease (AD) is a progressive neurological disorder characterized by loss of memory and cognition. *Stephania japonica* is being used as traditional medicine in the treatment of different neurological problems. In this study, we evaluated the anticholinesterase and antioxidant activities of the crude methanol extract of *S. japonica* and its fractions *in vitro* and the neuroprotective effect of the most active fraction in the scopolamine-induced mouse model of memory impairment. Among the crude extract and its fractions, chloroform fraction exerted strong inhibition of acetylcholinesterase and butyrylcholinesterase enzymes with IC_50_ values of 40.06 and 18.78 *µ*g/mL, respectively. Similarly, the chloroform fraction exhibited potent antioxidant activity and effectively inhibited the peroxidation of brain lipid in vitro. The phytochemical profile revealed the high content of polyphenolics and alkaloids in the chloroform fraction. Pearson's correlation studies showed a significant association of anticholinesterase and antioxidant activity with alkaloid and phenolic contents. Kinetic analysis showed that the chloroform fraction exhibited a noncompetitive type of inhibition. In experimental mice, the chloroform fraction restored the impaired learning and memory induced by scopolamine as evidenced by a significant decrease in latency time and increase of quadrant time in probe trial in Morris water maze task. The chloroform fraction also significantly reduced the activity of acetylcholinesterase and oxidative stress in mice. Our results suggest that the chloroform fraction of *S. japonica* may represent a potential candidate for the prevention and treatment of AD.

## 1. Introduction

Alzheimer's disease (AD) is commonly characterized by dementia deteriorating over the years and usually affects geriatric people. The pathological hallmarks of AD include significant deficiency of cholinergic neurons, the emergence of senile plaques comprising *β*-amyloid (A*β*) protein, and neurofibrillary tangles of microtubule-associated protein tau [1]. Oxidative stress and insufficient cholinergic functionality in the brain are considered two vital events in the development of AD [2, 3]. Despite the reasonable progress in perceiving the etiology and pathogenesis of AD in recent years, the effective drugs remain limited.

It is widely accepted that the cholinergic system that facilitates the deposition and retrieval of elements in memory is compromised in AD [4]. The diminished cholinergic transmissions along with depletion of acetylcholine (ACh) are held responsible for the generation and progression of dementia [5]. Thus, aiming to inhibit cholinesterase enzymes, which are responsible for hydrolyzing acetylcholine, has evolved as a major strategy in treating AD. Donepezil, galantamine, and rivastigmine are the only anticholinesterase agents approved by the US Food and Drug Administration for AD therapy till now. These drugs have been found to ameliorate the symptoms with improvement in the performance of AD patients; however, none of these drugs proved to be successful in restricting or reversing the development of the disease [6]. Therefore, explorations need to be continued for novel agents with potential efficacy and considerable safety.

Further, it has been revealed that oxidative stress plays a significant role in the pathogenesis of AD. The intervention of AD brain exhibited a high level of oxidative stress markers like reactive oxygen species (ROS), malondialdehyde (MDA), oxidized proteins, and DNA [7]. A mutual relation between oxidative stress and A*β* protein has been reported in several studies concluding the induction of oxidative stress by A*β* and elevated generation of A*β* by oxidative stress [8]. In fact, enhanced oxidative stress is hypothesized as an initial marker of AD pathogenesis contributing to further neuronal damage and cell death [3]. Therefore, the administration of potential antioxidative agents is suggested to minimize the ROS-mediated injury in AD.

Medicinal plants have been proved to be the potential source of remedies for various diseases, including AD. In Bangladesh, many plants have been used in traditional medicine to treat AD or related neurological disorders. Scientific evaluation of this medicine may lead to the development of new drugs or establishing them as alternative medicine. *Stephania japonica* (Thunb.) Miers, locally known as Foat pata, is a common scrambler belonging to the family Menispermaceae and grown throughout Bangladesh. The plant is traditionally used in the treatment of vertigo, headache, and sleep disturbances. The leaves are used to cure fever and pain and as a tonic [9, 10]. In Ayurveda and Sidha, the plant is being used in the treatment of various ailments, including bowel disorders, stomachache, dyspepsia, dysentery, diarrhoea, birth control, piles, urinary troubles, and heart troubles, and used as hypotensive and spasmolytic [11]. Biological investigations using *in vitro* and *in vivo* models have shown that the plant possesses multiple pharmacological effects, such as antioxidant, anti-inflammatory, cytotoxic/anticancer, analgesic, and hypoglycemic effects [12, 13]. Phytochemical analyses identified the hasubanan alkaloids as the major constituents along with phenolics and flavonoids, tannins, and steroids as minor constituents [13–15].

Although *S. japonica* has multiple therapeutic uses in traditional medicine, limited works have been done on its biological activity. Until now, there are no reports of the effect of this plant on neurological disorders. Therefore, the present study was designed to evaluate the crude methanol extract of *S. japonica* and its four solvent fractions for anticholinesterase and antioxidant activity *in vitro* and for neuroprotective effect in a scopolamine-induced mouse model of memory impairment.

## 2. Materials and Methods

### 2.1. Chemicals

Folin–Ciocalteu reagent, DPPH (1, 1′-diphenyl-2-picrylhydrazyl), aluminum chloride, potassium ferricyanide, and ammonium molybdate were procured from Sigma-Aldrich, India. 2-Deoxy-D-ribose, 2-thiobarbituric acid (TBA), bromocresol green (BCG), catechin, quercetin, 5,5′-dithio-bis-(2-nitro) benzoic acid (DTNB), acetylthiocholine iodide (ATCI), and S-butyrylthiocholine iodide (BTCI) were obtained from Sigma-Aldrich, Germany. All the employed chemicals were of analytical grade.

### 2.2. Plant Material

The stems of *Stephania japonica* were amassed from Rajshahi City, Bangladesh, in September 2018 and verified by an expert taxonomist (*Collection number: 369*). A voucher specimen was submitted for preservation in the herbarium affiliated with the Department of Botany, University of Rajshahi, Bangladesh.

### 2.3. Extraction

The coarse powder (1.2 Kg) of the stems was soaked in methanol for extraction at room temperature and kept for 7 days with occasional shaking and stirring. After filtration, the methanolic extract was concentrated in a rotary evaporator at reduced pressure to obtain the crude methanolic extract (CME, 85.7 gm). The resulting extract (50.0 gm) was partitioned with solvents of different polarity using the modified Kupchan method to yield the corresponding fractions: ethyl acetate (EAF, 2.41 gm), chloroform (CHF, 21.63 gm), petroleum ether (PEF, 5.16 gm), and aqueous (AQF, 10.26 gm) fractions [16]. All the fractions obtained were utilized for further chemical and biological experiments.

### 2.4. Phytochemical Screening of the Plant Extract

Qualitative tests were performed on the CME and its fractions to detect the presence of different phytochemicals, including phenolics, flavonoids, alkaloids, saponins, and phytosterols, using the standard phytochemical methods as described earlier [17].

#### 2.4.1. Estimation of Total Phenolic Content (TPC)

The TPC of the extract and fractions from *S. japonica* was determined by the Singleton method (1965) using Folin–Ciocalteu reagent (FCR) with gallic acid as standard [18]. Sample/standard solution (0.5 mL) was mixed with FCR (10 times diluted with distilled water, 2.5 mL) and Na_2_CO_3_ solution (7.5%, 2.5 mL) in the test tube and left at room temperature for 25 minutes in a dark place. The absorbance of the solution was measured at 760 nm. The result was calculated from the standard curve of gallic acid and expressed as mg of gallic acid equivalent (GAE)/g of dried extract.

#### 2.4.2. Estimation of Total Flavonoid Content (TFC)

The TFC of the extract and fractions from *S. japonica* was determined by aluminum chloride colorimetric method described by Dewanto et al. (2002) [19] using catechin as standard. Sample/standard solution (1 mL) was taken in the test tube, to which distilled water (5 mL) and NaNO_2_ (5% w/v, 0.3 mL) were added. After 5 minutes, AlCl_3_ (10% w/v, 0.6 mL) and NaOH (1M, 2 mL) were added to the mixture, and a final volume of 10 mL was made with distilled water. The reaction mixture was kept at room temperature for 30 minutes and then the absorbance of the solution was measured at 510 nm. The result was calculated from the standard curve of catechin and expressed as mg of catechin equivalent (CE)/g of dried extract.

#### 2.4.3. Estimation of Total Alkaloid Content (TAC)

The TAC of the extract and fractions from *S. japonica* was determined by the UV-Spectrophotometric method as reported by Ajanal et al. (2012) [20]. The extract/fraction was dissolved in 2 N HCl and filtered. Then 1 mL of this solution was transferred into a separating funnel, to which 5 mL of bromocresol blue solution and 5 mL of phosphate buffer (pH 4.7) were added. The mixture was shaken, and the complex formed was partitioned with chloroform. The fractions were collected in a volumetric flask and diluted to 10 mL with chloroform. The absorbance of the complex in chloroform was measured by a spectrophotometer at 470 nm. The result was calculated from the standard curve of atropine and expressed as mg of atropine equivalent (AE)/g of dried extract.

### 2.5. Study of In Vitro Activities

#### 2.5.1. Anticholinesterase Activity Tests

The cholinesterase inhibitory activity was assessed by the spectrophotometric method of Ellman et al. [21]. AChE from mice brain homogenate and BuChE from human blood was prepared by the procedure described earlier [17]. In brief, brain tissues were collected from mice, homogenized in 50 mM Tris-HCl (pH 7.4) containing 1.0 M NaCl and 50 mM MgCl_2_ and then centrifuged at 10,000 rpm for 20 minutes at 4°C to yield the crude AChE. To prepare the BuChE enzyme, human blood was collected in EDTA (1 mg/mL) treated test tube from an anonymous healthy male subject (24 years) at the Rajshahi University Medical Center, Rajshahi, Bangladesh, and centrifuged at 4000 rpm for 15 minutes at 4°C. The resulting plasma was used as the source of the BuChE enzyme. Acetylthiocholine iodide (ATCI) and S-butyrylthiocholine iodide (BTCI) were employed as substrates for AChE and BuChE enzymes, respectively. Hydrolysis of ATCI/BTCI by cholinesterase was monitored spectrophotometrically. 200 *µ*L of enzyme solution was mixed with 500 *µ*L of the test sample and kept for 20 minutes at 37°C. Just after adding Ellman's reaction mixture (3.5 mL; 0.5 mM acetylthiocholine iodide, 1 mM DTNB) in a phosphate buffer (pH 8.0, 50 mM), absorbance was recorded at 405 nm continuously for 5 minutes at 1 minute interval. A blank reaction was estimated by taking saline in place of the enzyme and a control reaction was also assessed by substituting the inhibitor with saline. Donepezil was used as a reference standard for AChE and galantamine for BuChE. The following formula was used to calculate the percentage inhibition of cholinesterase enzyme activity:

(1) $$\begin{array}{c} \%\text{ enzyme inhibition}=\frac{\left(\Delta A_{control}-\Delta A_{sample}\right)}{\Delta A_{control}}\times 100\%, \end{array}$$

where Δ*A*_*control*_ represents the change in absorbance per minute for control reaction; Δ*A*_*sample*_ represents the change in absorbance per minute for test sample reactions.

#### 2.5.2. Inhibition Kinetics of Cholinesterase Enzymes

The kinetic modes of AChE and BuChE inhibition by CHF were determined by preparing a range of CHF (inhibitor) concentrations (100, 200, and 400 *μ*g/mL) in which the concentration of the substrate (ATCI/BTCI) was varied (1.4, 0.7, 0.35, 0.175 and 0.0875 mM). With different concentrations (S) of the substrate (ATCI/BTCI), the velocity of the enzyme inhibition was different. Each assay was carried out three times. Lineweaver–Burk graph was plotted from S^−1^ vs. V^−1^ to determine the type of inhibition [22]. From these data, *V*_max_ (maximum reaction velocity) and *K*_m_ (dissociation constant) were calculated.

#### 2.5.3. Antioxidant Activity Tests

The antioxidant potential of the extract and fractions from *S. japonica* was tested by evaluating their DPPH (1, 1-diphenyl-2-picrylhydrazyl) free radical and hydroxyl radical scavenging activity, iron reducing power, lipid peroxidation inhibition, and total antioxidant capacity.

*DPPH (1, 1-Diphenyl-2-picrylhydrazyl) Free Radical Scavenging Assay.* The DPPH free radical scavenging activity of the extract and fractions from *S. japonica* was determined following the method described by Braca et al. (2001) [23]. The extract or standard at different concentrations (3.125, 6.25, 12.5, 25, 50, and 100 *µ*g/mL) was taken in test tubes, to which 3 mL of methanolic solution of DPPH (0.135 mM) was added. The tube was left at room temperature for 30 minutes in a dark place. The absorbance of the solution was measured at 517 nm using a spectrophotometer against a blank. A control reaction was also prepared with a methanolic solution of DPPH and other reagents instead of plant extract or standard solution. The percent (%) of DPPH radical scavenging was calculated from the following equation:

(2) $$\begin{array}{c} \%\text{ scavenging}=\frac{\left(A_{0}-A_{1}\right)}{A_{0}}\times 100\%. \end{array}$$

where *A*_0_ is the absorbance of the control; *A*_1_ is the absorbance of the extract/standard.

*Hydroxyl Radical Scavenging Assay.* Hydroxyl radical scavenging activity of the extract/fractions from *S. japonica* was determined following the method described by Halliwell and Gutteridge (1987) [24] with slight modification. In brief, a reaction mixture was prepared by taking 100 *μ*M FeCl_3_, 2.8 mM 2-deoxy-D-ribose, 20 mM potassium dihydrogen phosphate buffer (pH 7.4), 100 *μ*M EDTA, 1.0 mM hydrogen peroxide, and 100 *μ*M ascorbic acid. To 1 ml of the reaction mixture, sample (extract/standard) solution at different concentrations (12.5, 25, 50, 100, and 200 *µ*g/mL) was added and incubated for 1 hour at 37 °C. Afterward, 1 mL of the reaction mixture was added to 1.0 mL of 10% TCA and 1.0 mL of 1% TBA (in 0.1 M NaOH containing 0.025% BHA) and heated at 100°C for 20 minutes. The absorbance of the solution was measured spectrophotometrically at 532 nm against a blank solution. A control solution was prepared in the same way where all the reagents except the sample were present. The percent scavenging potential of samples was calculated from the following equation:

(3) $$\begin{array}{c} \%\text{ scavenging}=\frac{\left(A_{0}-A_{1}\right)}{A_{0}}\times 100\%. \end{array}$$

where *A*_0_ is the absorbance of the control solution and *A*_1_ is the absorbance of the extract samples and reference standard. All the tests were performed in triplicate.

*Ferric Reducing Power Assay.* The iron reducing power of the extract/fractions of *S. japonica* was evaluated by the method developed by Oyaizu (1986) [25]. A 2.5 mL sample/standard solution at different concentrations (12.5, 25, 50, and 100 *µ*g/mL) was added to 2.5 mL phosphate buffer (200 mM, pH 6.6) and 2.5 mL of 1% potassium ferricyanide and incubated at 50°C in a water bath for 20 minutes. After cooling, 2.5 mL of 10% trichloroacetic acid (TCA) was added to the mixture and centrifuged at 3000 rpm for 10 min. Then 5 mL supernatant was mixed with 5 mL of distilled water and 1 mL of ferric chloride and incubated at 37°C for 10 minutes. The absorbance of the mixture was measured at 700 nm against a blank solution.

*Lipid Peroxidation Inhibition Assay*. The inhibition of lipid peroxidation assay of the extract/fraction of *S. japonica* was determined according to the method described by Liu et al. (2000) with a slight modification [26]. Brain lipid was prepared from mouse brain through homogenization of brain in 50 mM phosphate buffer (pH 7.4) containing 0.15 M KCl and centrifugation at 10000 g for 15 min at 4°C. Sample/standard solutions at different concentrations (12.5, 25, 50, 100, and 200 *µ*g/mL) were mixed with 500 *μ*L of brain lipid and 0.2 mM FeCl_3_ and incubated at 37 °C for 30 minutes. After adding 2 ml of ice-cold HCl (0.25 N) containing 15% trichloroacetic acid (TCA), 0.38% TBA, and 0.5% BHT, the mixtures were heated at 80°C for 1 hour and centrifuged at 3000 rpm for 10 minutes to remove precipitated proteins. The solution was measured colorimetrically at 532 nm against a blank solution. A control solution was prepared in the same way that contained all the reagents except the sample. The percent inhibition of lipid peroxidation was calculated using the following formula:

(4) $$\begin{array}{c} \%\text{ inhibition}=\frac{\left(A_{0}-A_{1}\right)}{A_{0}}\times 100\%. \end{array}$$

where *A*_0_ is the absorbance of the control solution and *A*_1_ is the absorbance of the sample solution.

*Total Antioxidant Capacity Assay*. The total antioxidant capacity of the extract/fraction of *S. japonica* was determined by the method of Prieto et al. (1999) with some modifications [27]. Initially, 0.5 mL sample at different concentrations was added to 3 mL of the reaction mixture (0.6 M sulphuric acid, 28 mM sodium phosphate and 4 mM ammonium molybdate) in the test tube and heated at 95°C for 90 minutes in a water bath. After cooling, the absorbance of the reaction solution was measured at 695 nm against a blank.

### 2.6. In Vivo Study

The *in vivo* experiments were performed to assess the effects of CHF obtained from *S. japonica* stems employing a scopolamine-induced mouse model of memory impairment.

#### 2.6.1. Experimental Animals

Swiss Albino mice (male, 6 weeks) were collected from the International Center for Diarrhoeal Disease Research, Bangladesh (ICDDR, B) and kept in congenial conditions for seven days for the adjustment and suitable food and water were provided *ad libitum* according to the formula given by ICDDR, B. The experimental protocol was approved (*Ethical clearance number: 103*) by the Institutional Animal, Medical Ethics, Biosafety, and Biosecurity Committee (IAMBBC) at the Institute of Biological Science, University of Rajshahi. The behavioral assessments were usually conducted in the first half of the daytime.

#### 2.6.2. Plant Material and Treatment Schedule

Memory-enhancing potential of CHF was tested in scopolamine-induced memory-impaired mice using donepezil as a reference standard. All the drugs and test samples were dissolved in 6% dimethyl sulfoxide (DMSO)/94% water and injected intraperitoneally (4 mL/kg) into the mice. Thirty-six healthy mice were randomly selected and divided into six groups as follows:

  Group I: *normal vehicle group* (NVG), which received only DMSO (4 mL/kg).
  Group II: *scopolamine group* (SCO), which received scopolamine (1 mg/kg).
  Group III: *donepezil group* (DON), which received donepezil (5 mg/kg) [28] and scopolamine (1 mg/kg).
  Group IV–VI: *test groups* (CHF 100, 50, and 25), which received one of the three doses (100, 50, and 25 mg/kg) of the *S. japonica* CHF fraction and scopolamine (1 mg/kg).

**Figure 1:**
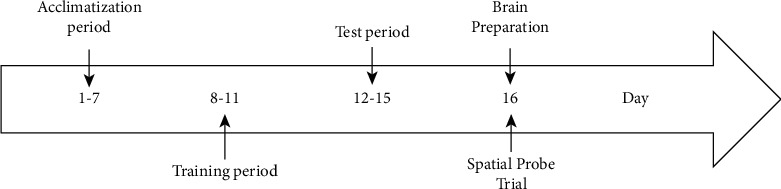
A general overview of the animal study timeline.

The experimental schedule is shown in Figure 1. Donepezil and CHF extract were administered to the mice of Groups III-VI during test days (Days 12–15) one hour before scopolamine injection. A resting period of thirty minutes was maintained after scopolamine administration to the mice before initiating behavioral assessments.

#### 2.6.3. Spatial Memory and Learning Test in Morris Water Maze

Effects of CHF on spatial memory and learning were investigated by employing the widely accepted Morris water maze (MWM) task in the scopolamine-induced mouse model of memory impairment [29, 30]. The components of the MWM include a large round black water tank (radius = 75 cm, height = 60 cm) and a 28 cm black platform. The water is maintained at room temperature with the platform constantly placed 1 cm under the water surface in the center of the northeast (NE) quadrant of the tank. After an acclimatization period of seven days, all the mice were trained for four consecutive days in the MWM and then tested for their spatial memory and learning for the next four days (Figure 1). During the training period (Days 8–11) of the mice, the highest 60 seconds was allotted for finding the concealed platform and 20 seconds to rest on it. Two trials were conducted daily for all mice. Throughout the test period (Days 12–15), the mean latency time (MLT) of all mice for each day was calculated from the two trials, where a substantial reduction in MLT indicates successful learning. On the next day (Day 16), the mice were made to go through a probe trial where the platform remained absent with an objective to test the spatial memory of the location of the platform. The mice were allowed to browse throughout the tank for 60 seconds and the time spent in the NE quadrant (last location of the platform) was noted. Higher time resided in the NE quadrant represents better performance.

#### 2.6.4. Biochemical Assays

Surgical decapitation was performed in mice on the 16^th^ day of the animal study program after being anesthetized with sodium pentobarbital (30 mg/kg; intraperitoneal injection). The mice brains were separated and homogenized in the extraction buffer (50 mM Tris-HCl buffer containing 1% Triton X-100) by a homogenizer. The resulting mice brain homogenate (MBH) was centrifuged at 5000 rpm for 20 minutes to obtain the supernatant and stored in the fridge for further experiments.

*Estimation of Protein Concentration*. The protein concentration of brain homogenate was estimated by the Lowry et al. (1951) procedure employing bovine serum albumin calibration curve [31]. The results were calculated as mg of protein present per mL of mice brain homogenate (MBH).

*Determination of Brain AChE Activity*. The AChE enzyme activity in mice brain homogenate was determined by Ellman et al. method (1961) as described earlier [21]. 200 *µ*L mice brain homogenate (MBH) was added to 2.2 mL 50 mM Tris-HCl buffer and 200 *µ*L DTNB (15 mM) and incubated for 15 minutes at room temperature. Then a change in absorbance per minute was noted at 412 nm immediately after adding 400 *µ*L acetylthiocholine iodide (3.75 mM) to the mixture. The specific enzyme activity was expressed as mU/mg of protein in MBH.

*Brain Reduced Glutathione (rGSH) Level*. rGSH level was estimated in MBH according to the method of Ellman et al. (1959) [32]. 0.25 ml MBH was mixed with 1.25 ml phosphate buffer (0.1 M, pH 7.2) and 1.5 ml distilled water. Then 20 *µ*L DTNB was added to the mixture and incubated for 1 hour at room temperature. The absorbance of the mixture was read at 412 nm. The amount of reduced glutathione was calculated with the Beer–Lambert formula using the extinction coefficient value of 13,600 M^−1 ^cm^−1^. The results are expressed as *μ*mol rGSH per mg of protein in MBH.

*Brain Malondialdehyde (MDA) Level*. MDA level in MBH was determined by the method described by Esterbauer and Cheeseman (1990) [33]. 0.25 ml MBH was mixed with 2.75 ml trichloroacetic acid (TCA, 10% w/v) and centrifuged to discard the protein pellets. The resulting supernatant was mixed with 3 ml TBA (0.67% w/v) and heated at 100 °C for 10 minutes in a water bath. The absorbance of the solution was measured at 532 nm. The amount of MDA was calculated using Beer–Lambert's formula with the extinction coefficient value of 1.56 × 10^5^ M^−1 ^cm^−1^. The result is expressed as nmol MDA per mg protein in MBH.

*Brain Superoxide Dismutase (SOD) Activity*. SOD activity was determined by the method described earlier [34], based on the ability of the enzyme to inhibit the autoxidation of pyrogallol. 50 *µ*L MBH was added to 1 ml Tris-EDTA buffer and then the change in absorbance per minute was noted immediately after adding 1 ml pyrogallol at 420 nm against Tris-EDTA buffer as blank. On the other hand, for the control reaction, the MBH was substituted with distilled water. SOD activities are expressed as units/mg of protein in MBH. One unit of SOD activity is defined as the amount of enzyme required to cause 50% inhibition of pyrogallol autoxidation [35].

*Brain Catalase (CAT) Activity*. CAT activity was measured by the method of Aebi et al. (1974) [36]. 0.1 mL MBH was added to the cuvette containing 1.9 mL of 50 mM phosphate buffer (pH 7.0). The reaction was started by adding 1.0 mL of freshly prepared 30 mM H_2_O_2_. The rate of decomposition of H_2_O_2_ by catalase was measured spectrophotometrically from changes in absorbance at 240 nm. A control reaction was also devised with the MBH replaced with 50 mM phosphate buffer (pH 7.0). The results were calculated using the molar extinction coefficient of 43.6 M^−1 ^cm^−1^ for hydrogen peroxide and expressed as units/mg protein in MBH.

### 2.7. Characterization of the CHF

Thin layer chromatographic (TLC) analysis of CHF was performed on TLC Silica gel 60 F_254_ Aluminum sheets (MERCK, Germany) with n-hexane:chloroform:methanol (4 : 4 : 2) as the mobile phase. UV spectra of the CHF were recorded in methanol solution using the BECKMAN double beam spectrophotometer in the Central Science Laboratory, University of Rajshahi, and the data are given in *λ*_max_. IR spectra of the CHF were recorded using the PERKIN ELMER 1600 FTIR spectrophotometer in the Central Science Laboratory, University of Rajshahi, and the data are given in cm^−1^.

### 2.8. Statistical Analysis

All output data were expressed as mean ± SD. Graph Pad Prism (version 8.0.1), SPSS (IBM SPSS Statistics 21.0), and Microsoft Excel 2010 were used for the statistical and graphical evaluations. The escape latencies in the Morris water maze task were analyzed by two-way ANOVA. The probe trial data in the water maze, biochemical parameters, and the data from in vitro assays were analyzed by one-way ANOVA. A *p*-value of **<** 0.05 was considered as significant artistically. IC_50_ values of different fractions/extractives were calculated using nonlinear regression (Dose-Response--Inhibition equation; log_10_ (inhibitor) vs. normalized response--variable slope) in Graph Pad Prism-8.0.1. A correlation study was performed using the Pearson correlation test.

## 3. Results

### 3.1. Phytochemical Analyses

Qualitative analyses of CME of *S. japonica* and its fractions indicated the presence of phenolics, flavonoids, alkaloids, saponins, steroids, and glycosides. Interestingly, all these phytoconstituents were found in the different fractions, but phenolics, flavonoids, and alkaloids were high in the CHF (Supplementary Table S1). Quantitative investigations revealed the highest content of phenolics (313.06 ± 2.76 mg GAE/g dried extract), flavonoids (167.25 ± 1.34 mg CE/g dried extract), and alkaloids (216.97 ± 4.63 mg AE/g dried extract) in the CHF followed by EAF, AQF, and PEF (Table 1).

TPC, total phenolic content; TFC, total flavonoid content; TAC, total alkaloid content; GAE, gallic acid equivalent; CE, catechin equivalent; AE, atropine equivalent; CME, crude methanolic extract, CHF, chloroform fraction; EAF, ethyl acetate fraction; AQF, aqueous fraction; PEF, petroleum ether fraction; values are expressed as mean ± standard deviation.

### 3.2. In Vitro Anticholinesterase Activity

#### 3.2.1. Anti-AChE and Anti-BuChE Activity

**Figure 2:**
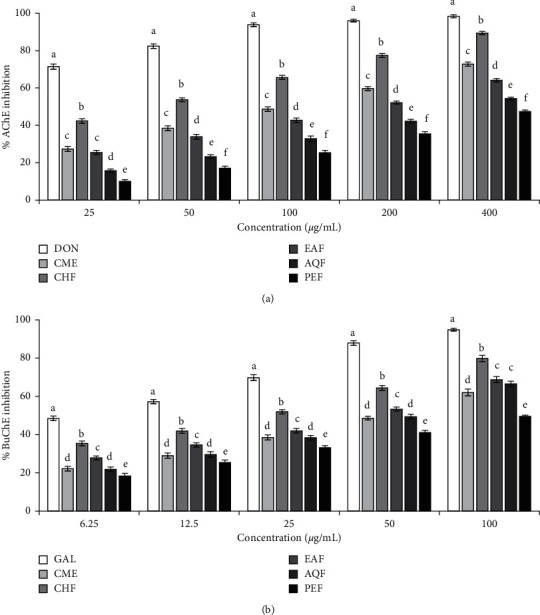
*Anticholinesterase activity of the crude methanol extract of Sjaponica and its fractions*. (a) AChE inhibitory activity. IC_50_ (*µ*g/mL): DON, 5.21 ± 0.18; CME, 104.13 ± 1.86; CHF, 40.06 ± 0.79; EAF, 159.57 ± 2.57; AQF, 308.57 ± 2.27; PEF, 453.17 ± 4.28. (b) BuChE inhibitory activity. IC_50_ (*µ*g/mL): GAL, 7.91 ± 0.35; CME, 50.44 ± 1.10; CHF, 18.78 ± 0.69; EAF, 35.30 ± 0.92; AQF, 44.73 ± 1.40; PEF, 100.08 ± 2.12. Results are expressed as mean ± SD (*n* = 3). Means with different letters (a–f) differ significantly (*P* < 0.05). CME, crude methanolic extract; CHF, chloroform fraction; EAF, ethyl acetate fraction; AQF, aqueous fraction; PEF, petroleum ether fraction; AChE, acetylcholinesterase; BuChE, butyrylcholinesterase; DON, donepezil; GAL, galantamine.

The inhibitory activities of the CME and its fractions against AChE and BuChE are presented in Figure 2. All the extract and fractions exhibited a dose-dependent inhibition of AChE and BuChE enzymes. The highest activity was found in the CHF followed by EAF, AQF, and PEF. The IC_50_ values of CHF were 40.06 ± 0.79 *μ*g/mL and 18.78 ± 0.69 *μ*g/mL for AChE and BuChE, respectively, suggesting that the CHF has greater inhibitory activity against BuChE than that of AChE. The anti-ChE activity of CHF was found to be good when compared with the strong cholinesterase inhibitor galantamine (for anti-BuChE) or donepezil (for anti-AChE) used in this study.

#### 3.2.2. Analysis of Mode of Inhibition

**Figure 3:**
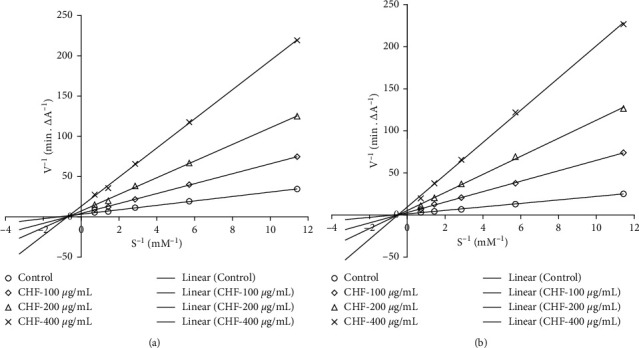
Lineweaver–Burk plot for inhibition of AChE (a) and BuChE (b) by different concentrations of CHF. Results represent the average values (*n* = 3).

Since CHF showed strong inhibition against both the AChE and BuChE, we investigated further to determine the modes of enzyme inhibition of this fraction using Lineweaver–Burke plots. Plots of AChE and BuChE inhibition by CHF were linear and intersected at a point on *X*-axis (Figure 3). These results indicated that CHF is a noncompetitive inhibitor for both AChE and BuChE.

### 3.3. In Vitro Antioxidant Activity

**Figure 4:**
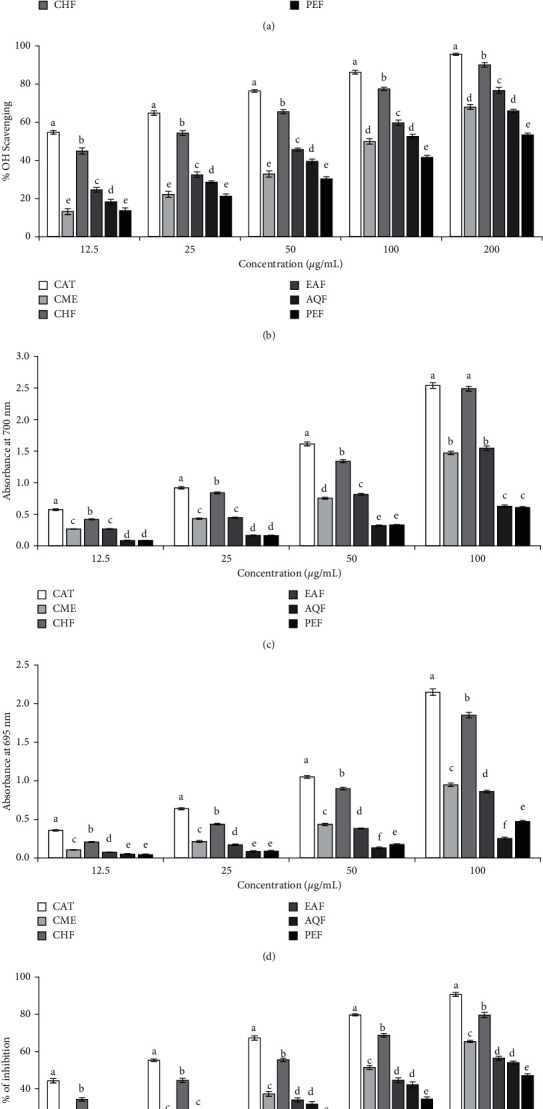
*Antioxidant activity of CME of S japonica and its fractions*. (a) DPPH radical scavenging activity. IC_50_ (*µ*g/mL): CAT, 3.03 ± 0.15; CME, 24.12 ± 1.00; CHF, 5.01 ± 0.16; EAF, 19.10 ± 0.73; AQF, 44.57 ± 1.90; PEF, 48.48 ± 3.47. (b) Hydroxyl radical scavenging activity. IC_50_ (*µ*g/mL): CAT, 9.00 ± 0.34; CME, 97.24 ± 1.09; CHF, 17.12 ± 0.23; EAF, 56.67 ± 0.63; AQF, 86.08 ± 1.88; PEF, 162.00 ± 1.76. (c) Ferric reducing power. At 100 *µ*g/mL concentration, the absorbances are as follows: CAT, 2.539 ± 0.045; CME, 1.471 ± 0.027; CHF, 2.489 ± 0.039; EAF, 1.548 ± 0.033; AQF, 0.627 ± 0.020; PEF, 0.609 ± 0.014. (d) Total antioxidant capacity. At 100 *µ*g/mL concentration, the absorbances are as follows: CAT, 2.147 ± 0.042; CME, 0.948 ± 0.022; CHF, 1.852 ± 0.035; EAF, 0.862 ± 0.018; AQF, 0.253 ± 0.016; PEF, 0.473 ± 0.016. (e) Lipid peroxidation inhibition. IC_50_ (*µ*g/mL): CAT, 18.23 ± 0.54; CME, 92.91 ± 2.35; CHF, 33.42 ± 0.45; EAF, 137.70 ± 1.31; AQF, 157.63 ± 3.15; PEF, 248.20 ± 2.50. Catechin (CAT) was used as a reference standard. Results are expressed as mean ± SD (*n* = 3). Means with different letters (a–f) differ significantly (*P* < 0.05). CAT, catechin; CME, crude methanolic extract; CHF, chloroform fraction; EAF, ethylacetate fraction; AQF, aqueous fraction; PEF, petroleum ether fraction.

Antioxidant activity of the CME and its fractions were assessed by several *in vitro* assays such as DPPH radical scavenging and hydroxyl radical scavenging, ferric reducing power, total antioxidant activity, and lipid peroxidation inhibition assay, and the results have been presented in Figure 4. The results of DPPH and hydroxyl radical scavenging revealed that all the extract and fractions have scavenging activity and the activity was found to be dose-dependent. The highest activity was found in CHF followed by EAF, AQF, and PEF. The IC_50_ values of CHF were 5.01 *μ*g/mL and 17.12 *μ*g/mL for DPPH and hydroxyl radical scavenging, respectively. Further, the activity of the CHF appeared to be good when compared with the strong antioxidant catechin, which showed an IC_50_ value of 3.03 *μ*g/mL and 9.00 *μ*g/mL for DRS and HRS, respectively.

In ferric reducing power assay and total antioxidant capacity assay, CHF also exhibited the highest antioxidant activity, followed by EAF, AQF, and PEF. The absorbance values of CHF at 100 *μ*g/mL concentration were 2.49 and 1.85 for ferric reducing power and total antioxidant capacity, respectively.

In lipid peroxidation inhibition assay, peroxidation of lipid was induced in the brain homogenate by hydroxyl radical generated in Fenton reaction and the ability of the CME and its fractions to inhibit the peroxidation of lipid was then assessed. The result is shown in Figure 4(e). As expected, CHF exhibited the highest activity among the fractions with an IC_50_ of 33.42 *μ*g/mL and PEF, the lowest with an IC_50_ of 248.20 *μ*g/mL. The overall results indicated the superior antioxidant potential of CHF of *S. japonica*.

### 3.4. Correlation between Phytochemicals and Anticholinesterase and Antioxidant Activity

The bioactivity of a plant is mediated by its phytochemicals. Since the CME of *S. japonica* and its fractions were found to contain a considerable amount of polyphenolics and alkaloids and exhibited potent antcholinesterase and antioxidant activities, we tested the correlations between these phytochemical contents and cholinesterase inhibitory and antioxidant activity and the results are shown in Table 2. A significant correlation (*P* < 0.05) was observed between TAC and BuChE inhibition (*r* = 0.961), hydroxyl radical scavenging (*r* = 0.979), and lipid peroxidation inhibition (*r* = 0.939). Likewise, the correlation between TPC and AChE inhibition (*r* = 0.920), DPPH radical scavenging (*r* = 0.943), lipid peroxidation inhibition (*r* = 0.928), ferric reducing power (*r* = 0.971), and total antioxidant capacity (*r* = 0.963) was also found to be significant (*P* < 0.05). The TFC showed a significant association with DPPH radical scavenging (*r* = 0.929) and ferric reducing power (*r* = 0.931). These results suggested the involvement of alkaloids and polyphenolics in the inhibition of cholinesterase and antioxidant activity.

### 3.5. Effect of CHF on Spatial Memory and Learning in the Morris Water Maze Task

**Figure 5:**
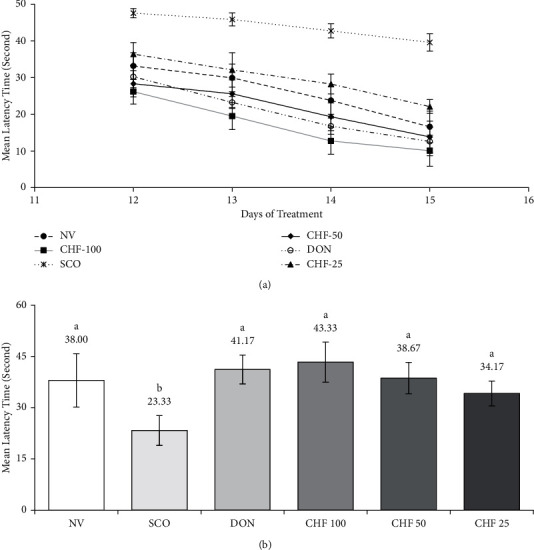
Effect of CHF on scopolamine-induced learning and memory impairments in mice by Morris water maze (MWM) task. (a) Changes in the mean latency time during 4 consecutive days of training for mice of different groups in the MWM task. (b) Average time spent in the platform quadrant by mice of different groups in the MWM probe trial. Each group was significantly different (*P* < 0.05) from the disease control group (scopolamine-treated group). Values were expressed as MLT ± STD, *n* = 6 mice for each group.

The time taken by mice of different groups to find the platform (escape latency) in the Morris water maze (MWM) task is shown in Figure 5(a). In the MWM test, the mean latency time (MLT ± SD) of the normal vehicle group (NVG) to the platform was 33.2 ± 3.59 sec on the 12th day, abated rapidly during the learning period of the next 3 days, and became 16.6 ± 4.32 sec on the 15th day. On the other hand, the MLT of the scopolamine (SCO) group decreased slightly with 47.6 ± 1.24 sec on the 12th day and 39.6 ± 2.33 sec on the 15th day, indicating the marked impairment of memory. In comparison, test groups induced with 100, 50, and 25 mg/kg of CHF and standard donepezil (DON) showed MLT of 26.2 ± 3.36, 28.3 ± 3.59, 36.4 ± 3.12, and 30.3 ± 3.03 sec, respectively, on the 12th day and 10.1 ± 4.21, 13.8 ± 4.32, 22.1 ± 1.88, and 12.6 ± 3.87 sec, respectively, on the 15th day of the treatment, suggesting the reversal of memory impairment induced by scopolamine (*P* < 0.05).

The times (mean ± SD) spent within the desired quadrant of the NVG, SCO, 100, 50, and 25 mg/kg of CHF, and DON groups during the probe trial are shown in Figure 5(b). The results showed that NVG, 100, 50, and 25 mg/kg of CHF, and DON groups spent more time in the desired quadrant in comparison to the SCO, indicating improvement of spatial memory by CHF in mice (*P* < 0.05).

### 3.6. Effect of CHF on Biochemical Parameters

#### 3.6.1. AChE Activity in Mouse Brain

**Figure 6:**
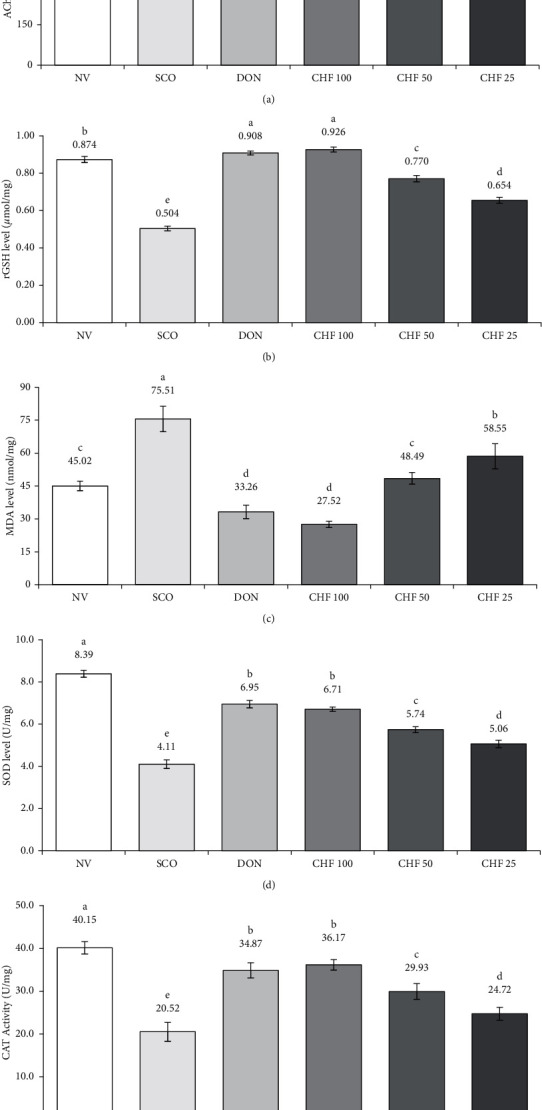
Effect of CHF on brain AChE activity and oxidative stress biomarkers in the scopolamine-induced mouse model. (a) AChE activity, (b) rGSH level, (c) MDA level, (d) SOD activity, and (e) CAT activity in the brain of different groups of mice. Means with different letters (a-f) differ significantly (*P* < 0.05), where all the drug-treated groups were significantly different (*P* < 0.05) from the disease control group (scopolamine-treated group). Values were expressed as mean ± STD, *n* = 6 mice for each group. AChE, acetylcholinesterase; NV, normal vehicle group; SCO, scopolamine; DON, donepezil; CHF, chloroform fraction; rGSH, reduced glutathione; MDA, malondialdehyde; CAT, catalase.

AChE activity in mice of the SCO group was increased by 2.13-fold in comparison to NVG. In contrast, administration of 100, 50, and 25 mg/kg of CHF inhibited the AChE activity significantly (*P* < 0.05) in scopolamine-induced mice in a dose-dependent manner, which was similar to that of DON (Figure 6(a)).

#### 3.6.2. rGSH and MDA Level in Mouse Brain

The level of rGSH was decreased significantly (*P* < 0.05) in the brain of SCO group mice compared to NVG. CHF of 100, 50, and 25 mg/kg increased significantly (*P* < 0.05) the level of rGSH in scopolamine-induced mice. On the other hand, the level of MDA was increased significantly (*P* < 0.05) as compared to NVG and CHF of 100, 50, and 25 mg/kg decreased significantly (*P* < 0.05) the level of MDA in scopolamine-induced mice The rGSH and MDA levels were reversed to almost normal by both CHF of 100 mg/kg and DON treatment (Figures 6(b) and 6(c)).

#### 3.6.3. SOD and CAT Activity in Mouse Brain

The activity of SOD and CAT was reduced significantly (*P* < 0.05) in the brain of the SCO group as compared to NVG. CHF of 100, 50, 25 mg/kg and DON increased (*P* < 0.05) significantly the SOD and CAT activity in scopolamine-induced mice (Figures 6(d) and 6(e)).

### 3.7. Characterization of CHF by TLC, UV, and IR Spectroscopy

**Figure 7:**
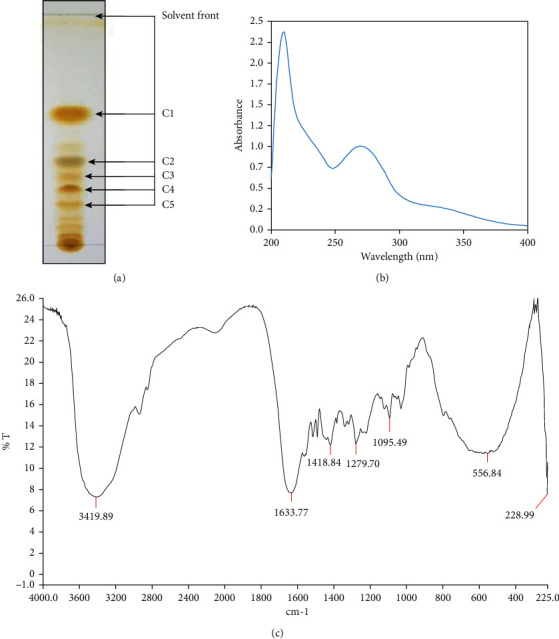
Chromatographic and spectroscopic characterization of the CHF. (a) Thin layer chromatography (TLC) profile, (b) UV spectrum, and (c) IR spectrum of CHF.

Since CHF showed a potential neuroprotective effect in mice, we used thin layer chromatography (TLC) and a combination of UV and IR spectroscopy to analyze the compounds in the fraction. TLC analysis showed five major compounds (C1 to C5) with *R*_*f*_ values of 0.581, 0.372, 0.302, 0.244, and 0.186 (Figure 7(a)). All the compounds were stained as positive in Dragendorff's reagent, indicating that they were alkaloid in nature. The UV spectrum (Figure 7(b)) of the fraction showed absorption maxima at 270 nm, indicating the presence of alpha, beta-unsaturated carbonyl functionalities. IR spectrum (Figure 7(c)) displayed characteristic absorption bands at 3420, 1634, 1419, 1280, and 1096 cm^−1^ attributable to hydroxyl and carbonyl groups as well as aromatic functionalities.

## 4. Discussion

AD is a neurological disorder of aged people clinically characterized by gradual loss of memory and cognition. Numerous etiologic factors have been recognized in AD, among which acetylcholine, A-beta, and tau proteins play crucial roles in the pathogenesis [1]. According to cholinergic hypothesis, the progressive decline of acetylcholine in AD is closely related to the loss of memory and cognition [4, 5]. This hypothesis has formed the basis for developing the current AD drugs. A-beta protein is thought to be central in the pathogenesis of AD as it is excessively generated in AD, undergoes aggregation, and results in neurotoxicity and neuronal damage. One of the neurotoxic pathways of A-beta is the generation of free radicals that damage all the biomolecules and produce oxidative stress [7, 8]. Oxidative stress has also been found to be associated with the loss of memory and cognition. Due to the multifactorial nature of AD, a multitargeted drug rather than a single targeted drug has now been suggested and explored as the therapeutics for AD [37]. In recent years, many efforts have focused on medicinal herbs, which are employed in traditional medicine to treat AD or related disorders. *Stephania japonica* is a perennial twining shrub used as the traditional medicine in the treatment of headache, vertigo, and sleep disturbances and as a tonic in Bangladesh [9, 10]. Herein, we report for the first time that *Stephania japonica* has potential anticholinesterase and antioxidant activities *in vitro* and *in vivo* and is able to ameliorate the scopolamine-induced deficits in learning and memory.

The crude methanol extract of *S. japonica* was fractionated into four different fractions such as PEF, CHF, EAF, and AQF for phytochemical and biological investigation. Preliminary analysis of phytochemicals revealed that the plant is rich in alkaloids and polyphenolics (phenolics and flavonoids), which were mostly found in the CHF (Supplementary Table S-1). Polyphenolics and alkaloids are common secondary metabolites of plants that display important biological activities. Polyphenolics are considered natural antioxidants due to the presence of hydroxyl group and have the ability to scavenge free radicals by donating electron or hydrogen. Alkaloids have been reported to exert multiple biological activities, including antioxidant and acetylcholinesterase inhibitory activity [37]. Quantitative analysis of polyphenolics and alkaloid content revealed that the CHF contained the highest content of phenolics (313.06 mg GAE/g dried extract), flavonoids (167.25 mg CE/g dried extract), and alkaloids (216.97 mg AE/g dried extract), followed by EAF, AQF, and PEF (Table 1). These results were in good agreement with the previous reports that showed the occurrence of phenolic hasubanan alkaloids as the major compounds in the CHF [13, 14]. The presence of a large amount of polyphenolics and alkaloids in the CHF and its high antioxidant and cholinesterase inhibitory properties suggest that they might be attributable to the bioactivity of the extracts.

AChE is a key component of the central cholinergic system and a primary enzyme involved in the hydrolysis of ACh into acetic acid and choline. Like AChE, BuChE also hydrolyzes acetylcholine, but to a lesser extent. Inhibition of AChE and BuChE has been found to increase the endogenous level of ACh neurotransmitter, elevate the cholinergic neurotransmission in the brain, and result in improvement of learning and memory [17, 38]. In this investigation, we found that all the tested extracts and fractions exhibited a dose-dependent inhibition of AChE and BuChE enzymes (Figure 2). Among them, CHF showed the highest activity with IC_50_ of 40.06 and 18.78 *µ*g/mL for AChE and BuChE, respectively. The lower IC_50_ value of the fraction against the BuChE than that against AChE suggests that the fraction has a higher specificity for BuChE than that of AChE. The TPC, TFC, and TAA contents were high in the CHF. Pearson correlation showed a significant association between TAC and BuChE inhibition and between TPC and AChE inhibition (Table 2). We also investigated the inhibition kinetics of the chloroform fraction against both AChE and BuChE by using a Lineweaver–Burk plot. The Lineweaver–Burk plot (Figure 3) showed that all the data lines intersect at a point on *X*-axis, indicating the noncompetitive inhibition of both AChE and BuChE.

Oxidative stress has recently been recognized as a common pathway of neurotoxicity in many neurodegenerative diseases, including AD. It is extensive in AD and play roles in the progression and development of AD. Antioxidants have been shown to prevent oxidative stress-induced damage and improve memory in the animal models of AD [37, 39]. In this study, we have assessed the antioxidant activity of CME and its fractions using several antioxidant assays such as DPPH and hydroxyl radical scavenging, reducing power, and total antioxidant activity. In all assays, CHF showed the highest activity compared to the other extract/fractions (Figure 4). The antioxidant activity of the CHF appeared to be strong when compared to the reference standard catechin. The IC_50_ of the CHF for DPPH and hydroxyl radical scavenging was 5.01 and 17.12 *µ*g/mL, while it was 3.03 and 9.00 *µ*g/mL for catechin. Similarly, the reducing activity and total antioxidant activity of the CHF were 2.49 and 1.85 at 100 *µ*g/mL, while they were 2.54 and 2.15 for catechin. The antioxidant activity was also reflected in lipid peroxidation inhibition. CHF effectively inhibited the lipid peroxidation with an IC_50_ value of 33.42 *µ*g/mL. In many plants, the antioxidant activity has been found to be correlated with the phenolics and flavonoids content [39]. We also found a strong correlation between TPC and DRS, LPI, FRP, and TAC and between TAC and HRS and LPI (Table 2). The cholinesterase inhibitory and antioxidant activities of the CHF suggest that it might be effective in preventing or reducing the impairment of memory caused by cholinergic deficit and oxidative stress in AD.

The protective effect of the most potent CHF on impairment of learning and memory was evaluated in the scopolamine-induced mouse model of memory impairment. We chose this model as it recapitulates the important features of AD, like oxidative stress, cholinergic deficit, and toxicity [30, 40]. Scopolamine affects the cholinergic function by inhibiting muscarinic receptor and impairs memory and function. The improvement of learning and memory was measured by the Morris water maze task. In our study, we found a significant increase of the mean latency time in the 4-day treatment with scopolamine compared with the control, indicating that cognitive impairment was induced by scopolamine (Figure 5(a)). Scopolamine also reduced the latency time spent in the probe (target quadrant) as compared with the control group (Figure 5(b)). These results were in accordance with that of the previous works [38]. The mean latency and time spent in the probe were reversed to normal by the CHF and the reference compound donepezil. Our results demonstrated the therapeutic effect of CHF in the improvement of learning and memory.

The mechanisms by which the CHF ameliorated the impairment of memory and cognition in scopolamine-induced mice were explored. It has been reported that the impairment of memory in the scopolamine-induced animal model is associated with cholinergic dysfunction and oxidative stress [40]. Scopolamine has been found to induce an increased level of AChE and oxidized molecules such as MDA and rGSH and reduction of antioxidative enzymes such as SOD and CAT in the brain of model mice, which are considered as oxidative stress biomarkers. In this study, we have measured the level of AChE and oxidative stress markers in the brain of scopolamine-treated mice. There was a significant increase in AChE activity in mice treated with scopolamine compared with normal mice (Figure 6(a)). These results were consistent with the results reported earlier [38]. The increase of AChE was reversed to a normal level by treatment of CHF and donepezil. Similar to AChE, there was a significant increase of rGSH and MDA levels (Figures 6(b) and 6(c)) and reduction of SOD and CAT levels when compared with the control group (Figures 6(d) and 6(e)). These effects were reversed by treatment with the CHF and donepezil, suggesting that the CHF is capable of reducing the oxidative stress in scopolamine-treated mice. Taken together, our results indicate that the CHF contributes to improving the ability of learning and memory through inhibition of AChE and oxidative stress.

To gain insights into the compounds responsible for the activity, we investigated further the CHF by thin layer chromatography (TLC) and a combination of UV and IR spectroscopy. TLC profile of the CHF showed five distinct compounds (Figure 7(a)) and they were positive in Dragendorff's reagent, suggesting that the compounds were alkaloid in nature. UV and IR spectrum of the fraction showed the presence of hydroxyl and alpha, beta-unsaturated carbonyl groups and aromatic functionalities (Figures 7(b) and 7(c)). Importantly, the UV and IR spectrum of the chloroform fraction appears very similar to that of hasubanan alkaloids, as reported earlier [41]. A literature review has shown hasubanan alkaloids as the major compounds isolated from this plant and nearly 50 alkaloids of this class have been reported from different plants of the genus [13, 14]. Based on the TLC profile, UV and IR spectrum of the CHF and a comparison with the previously isolated compounds from this plant suggest that the hasubanan alkaloids are possibly the major compounds of the CHF, which might be involved in the cholinesterase inhibition and antioxidant activity. Further works on isolation and characterization of the active compounds in the chloroform fraction are underway.

## 5. Conclusion

In summary, the results of this study clearly demonstrate that the chloroform fraction of *Stephania japonica* possesses potential anticholinesterase and antioxidant activities *in vitro* and *in vivo* and is able to ameliorate the scopolamine-induced deficits in learning and memory. Our data suggest that the chloroform fraction may represent a promising candidate for treating Alzheimer's disease. To the best of our knowledge, this is the first report of antioxidant, anticholinesterase, and memory-enhancing potentials of *S. japonica*. With further validation, the chloroform fraction of *S. japonica* holds a great optimism to be useful in both the prevention and treatment of Alzheimer's disease.

## Figures and Tables

**Table 1:** Quantitative phytochemical analysis of different extracts of *S. japonica*.

Samples	TPC	TFC	TAC
	mg GAE/g dried extract	mg CE/g dried extract	mg AE/g dried extract
CME	212.36 ± 3.60	90.47 ± 2.27	111.73 ± 3.10
CHF	313.06 ± 2.76	167.25 ± 1.34	216.97 ± 4.63
EAF	149.22 ± 3.40	134.99 ± 1.62	129.19 ± 2.65
AQF	61.94 ± 3.18	78.22 ± 2.91	115.70 ± 2.15
PEF	51.77 ± 3.42	71.76 ± 1.62	13.63 ± 2.62

**Table 2:** Pearson correlation coefficients of total phenolic, flavonoid, and alkaloid contents with cholinesterase inhibitory and antioxidant activities.

	TPC	TFC	TAC
AChEI	0.919	0.778	0.877
BuChEI	0.713	0.780	0.961
DRS	0.943	0.929	0.863
HRS	0.765	0.888	0.979
LPI	0.927	0.762	0.939
FRP	0.970	0.931	0.859
TACA	0.963	0.885	0.786

AChEI, acetylcholinesterase inhibition; BuChEI, butyrylcholinesterase inhibition; DRS, DPPH radical scavenging; HRS, hydroxyl radical scavenging; LPI, lipid peroxidation inhibition; FRP, ferric reducing power, TACA, total antioxidant capacity.

## Data Availability

The data used to support the findings of this study are available from the corresponding author upon request.

## Supplementary Materials

Supplementary Materials Table S1: qualitative phytochemical screening of different extracts from *S. japonica*. .
