# Supplementary material for: Stephania japonica Ameliorates Scopolamine-Induced Memory Impairment in Mice through Inhibition of Acetylcholinesterase and Oxidative Stress
Source: Adv Pharmacol Pharm Sci. 2022 Feb 21;2022:8305271. doi: 10.1155/2022/8305271 (PMC8885280; doi:10.1155/2022/8305271)
Supplement: Supplementary Materials — Table S1: qualitative phytochemical screening of different extracts from S. japonica. . [file 8305271.f1.docx]

**Table S1: Qualitative phytochemical screening of different extracts from *S. japonica***

| Phytoconstituents | **CME** | **CHF** | **EAF** | **AQF** | **PEF** |
| --- | --- | --- | --- | --- | --- |
| Phenolics | **++** | **+++** | **++** | **+** | **+** |
| Flavonoids | **++** | **+++** | **++** | **+** | **+** |
| Alkaloids | **++** | **+++** | **++** | **++** | **+** |
| Phytosterols | **++** | **+** | **+** | **-** | **++** |
| Tannins | **+** | **++** | **+** | **+** | **-** |
| Saponins | **+** | **-** | **+** | **+** | **-** |

Here, + = Present in small quantity, ++ = Present in modest amount, +++ = Present in large quantity, − = Absence.
